# Botryoid Wilms’ tumor: a case report and review of the literature

**DOI:** 10.1186/1477-7819-11-102

**Published:** 2013-05-20

**Authors:** Guofeng Xu, Jimeng Hu, Yeming Wu, Yongtao Xiao, Maosheng Xu

**Affiliations:** 1 Department of Pediatric Surgery, XinHua Hospital Affiliated to Shanghai Jiao Tong University School of Medicine, 1665, Kongjiang Road, Shanghai 200092, P. R. China; 2 Shanghai Institute for Pediatric Research, 1665, Kongjiang Road, Shanghai 200092, P. R. China

**Keywords:** Wilms’ tumor, Botyroid Wilms’ tumor, Renal, Computed tomography, Diagnosis

## Abstract

Here, we report a new case of botryoid Wilms’ tumor, a 4-year-old boy, who was referred to us with a chief complaint of dysuria and gross hematuria. The computed tomography and radical nephroureterectomy showed that a botryoid sarcoma-like appearance occupied the right renal pelvis and extended into the bladder. Histologic examination further confirmed this case was a mixed type of Wilms’ tumor. In a word, we demonstrated a rare case of botryoid Wilms’ tumor, which extended from the renal pelvis into the ureter and bladder, then some degenerative and necrotic tissue with calcification discharged from urethra. Postoperative adjuvant chemotherapy was executed. At 24-month follow-up, there was no evidence of recurrence.

## Background

Wilms’ tumor is the most common renal neoplasm in children. Wilms’ tumor usually originates from the renal parenchyma and expands into the surrounding tissue. Botryoid Wilms’ tumor is a great rarity. The differential diagnosis might be challenging; it may depend on the gender, regional difference, and environmental exposure of the patient. Based on the case of a 4-year-old boy, we discuss the pathogenesis, diagnosis,treatment of this rare entity, and present a review of the still scarce published literature on the subject.

## Case presentation

**Figure 1:**
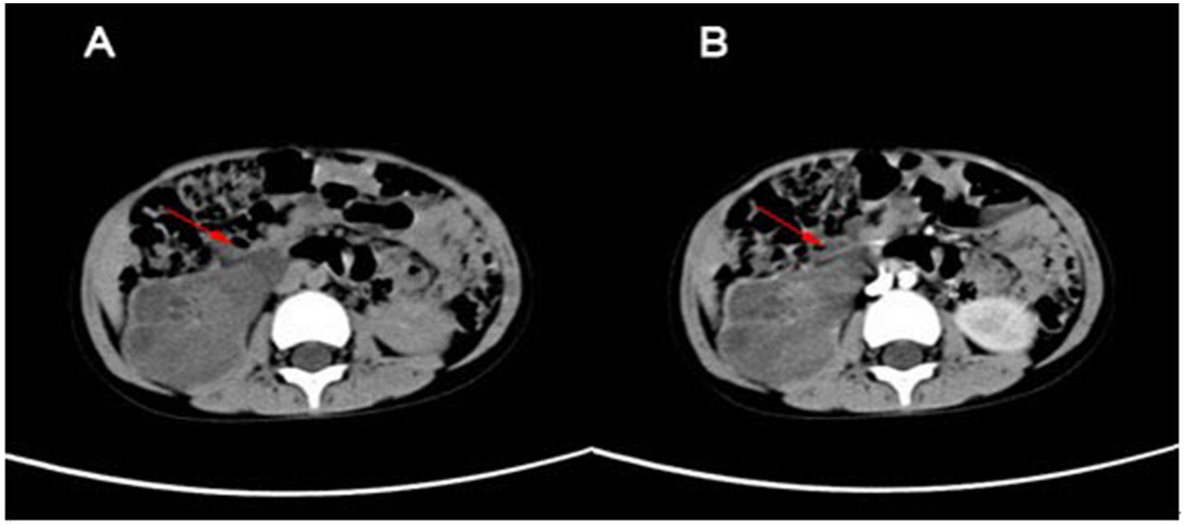
**Non-contrasted CT and Contrasted enhanced CT.** (**A**) Non-contrast CT illustrated enlarged inhomogeneous right kidney consisting of multiple water-density masses, which filled the pelvicalyceal system. (**B**) Contrasted-enhanced CT showed that the masses at the same level were enhanced slightly and inhomogeneously.

**Figure 2:**
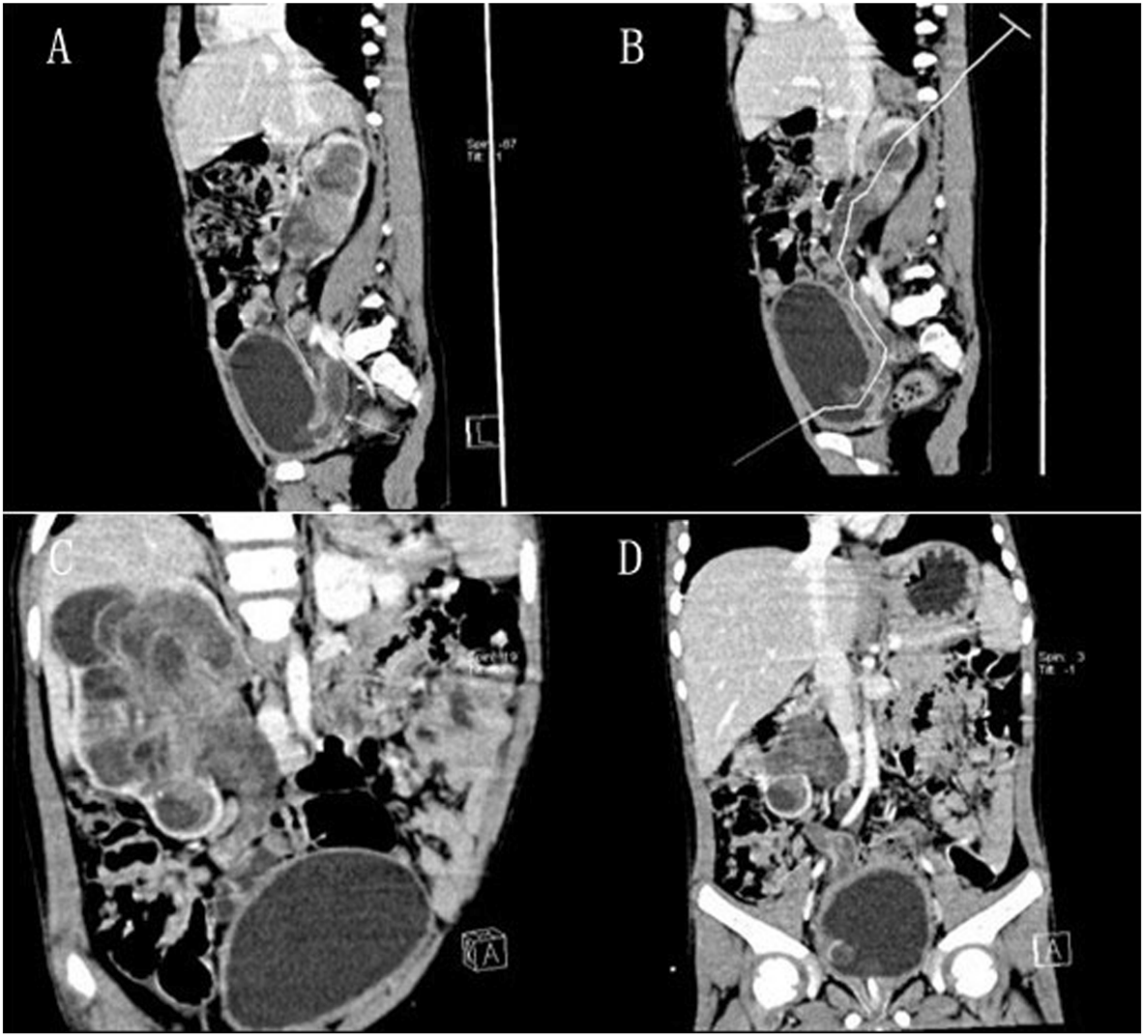
**Nephrogenic phase sagittal and coronal CT.** (**A**,**B**) Sagittal CT showed the botryoid tumor mass extended from the renal pelvis into the ureter and the bladder. (**C**,**D**) Coronal CT illustrated a botryoid sarcoma-like appearance.

**Figure 3:**
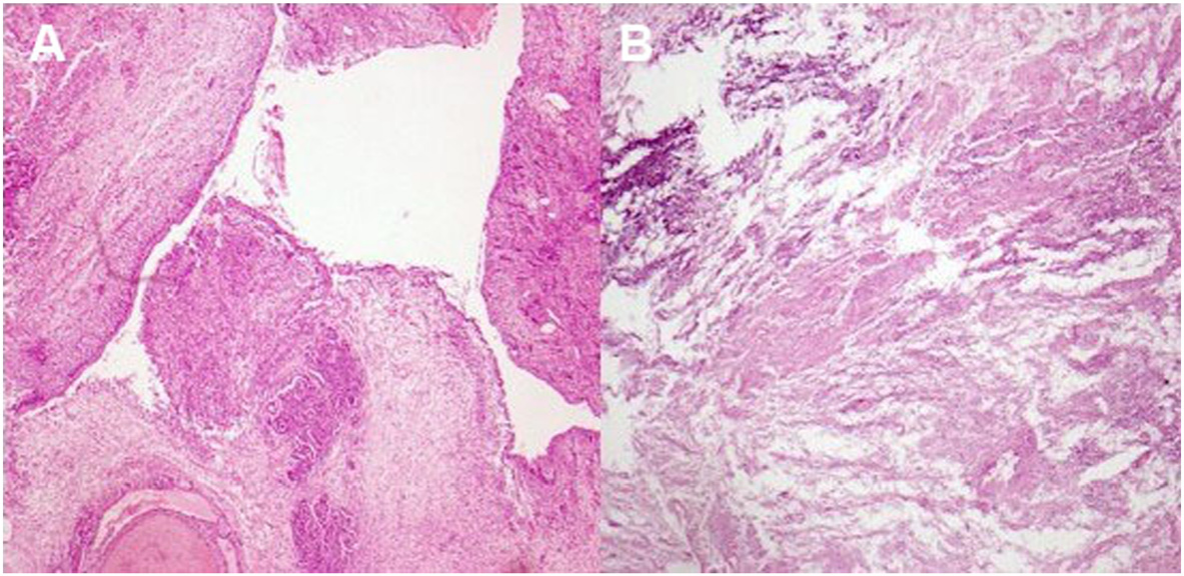
**Lesion characteristics under microscope.** (**A**) Microscopically, tumor extended into the pelvis by transitional cell of the urothelium. (**B**) The tissue discharged from urethra illustrated degenerative and necrotic tissue with calcification.

A 4-year-old boy was referred to our hospital with the chief complaint of dysuria and gross hematuria. Physical examination showed that there was a 10 × 8 cm solid, non-tender, and smooth surface mass in the hypogastrium. On blood analysis, the white blood cell (WBC) count was 11,770/mm3 (normal, 8,000/mm3 to 12,000/mm3) and C-reactive protein level was 6.0 mg/dL. His hemoglobin level was 11.7 mg/dL (normal, 11.0 mg/dL to 16.0 mg/dL). Urinalysis results showed numerous red blood cells per high-power field and white blood cells were negative. Renal and hepatic function, electrolytes examinations were normal (Na ^+^ 143 mmol/L, K ^+^ 5 mmol/L, ALT 6 u/L, AST 46 u/L, Cr 62.8 umol/L, BUN 9.1 mmol/L). An ultrasonography examination revealed that the right kidney, right ureter, and bladder were filled with heterogeneous-hypoechoic appearance, which indicated that the tumor had occupied right renal pelvis, ureter, and extended into the bladder. There was also left hydronephrosis and ureter dilated according to the ultrasonography examination. Abdominal computed tomography (CT) scan examination showed a right enlarged inhomogeneous renal mass (Figure 1A to 1B), tumor tissue mass that extended from the right renal pelvis, ureter into the bladder (Figure 2). During treatment, the catheter had been blocked up.After we pulled out the urethral catheter, some degenerative and necrotic tissue with calcification discharged from urethra (Figure 3B).

**Figure 4:**
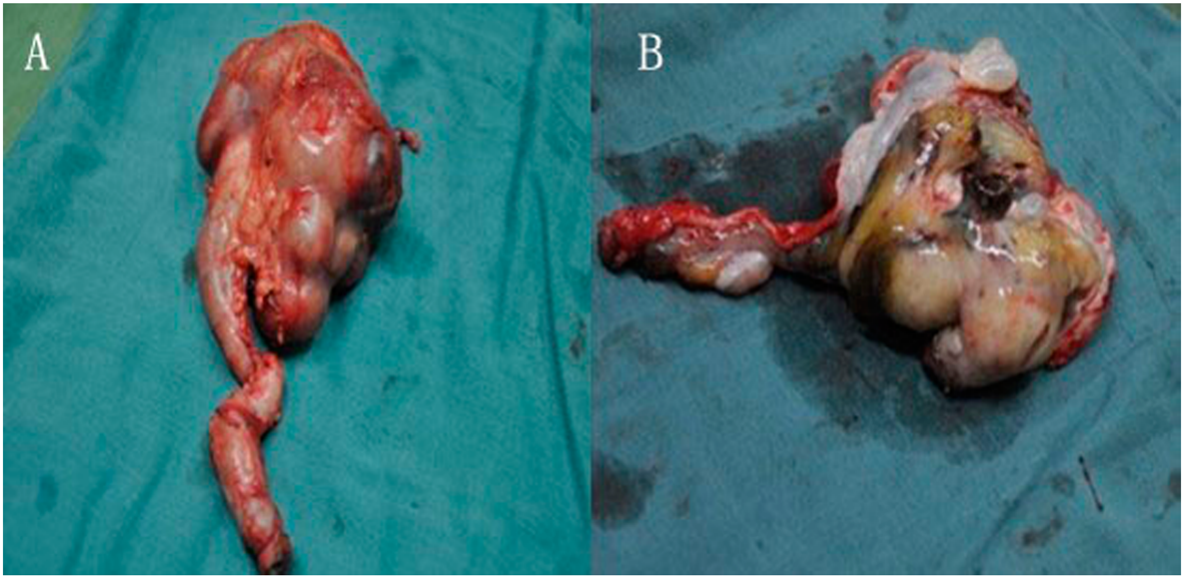
**Resected specimen.** (**A**) Botryoid sarcoma-like appearance, occupied the right renal pelvicaliceal system, renal pelvis and ureter. (**B**) Grayish polypoid mass with coagulation necrosis.

A diagnosis of Wilms’ tumor growing into the collecting system was confirmed, and a radical right nephroureterectomy was accomplished through a transabdominal approach. During the operation, we found a 10 × 8 × 6 cm grayish polypoid mass with coagulation necrosis occupying a large proportion of the renal pelvis and growing into the distal ureter (Figure 4A and 4B). The results of the pathological report revealed microscopically, the typical features of Wilms’ tumor with blastemal, epithelial, and stromal components were evident. The renal sinus, renal capsule, renal hilar lymph nodes, renal artery, and vein were free from tumor. Through the above clinical, imaging, and histologic findings, a stage II Wilms’ tumor was diagnosed. Postoperative adjuvant chemotherapy with dactinomycin and vincristine for stage II were executed referring to the regimen of the National Wilms’ Tumor Study Group 5 (NWTSG-5). At the time of writing this paper, there was no local recurrence or metastatic occur after the surgery for 2 years.

## Discussion

Wilms’ tumor is the most common malignant renal neoplasm occurring during childhood. It usually originates from the renal parenchyma and germinates by expanding into the surrounding tissue. In rare circumstances, the Wilms’ tumor expands into the renal collecting system and has an appearance similar to botryoid sarcoma. Thus, such tumors are called botryoid Wilms tumor. For the clinical manifestations that distinct from typical Wilms tumor, an asymptomatic mass is the most common clinical feature of a typical Wilms’ tumor, with other features happening in <25% of cases [1-7].

To the best of our knowledge, only 20 cases of botryoid Wilms’ tumor have been reported in the literature (Table 1). In these 20 cases of botryoid Wilms’ tumor reported in the literature (14 male cases, six cases), with 14 cases occurring in right side urinary system and six cases occurring in the left. It seems that the incidence of BWT is much higher in boys than in girls. Ten of 20 cases reported are from East Asia. Gender and regional difference, environmental factor may be important pathogenic elements of BWT. Presently, the pathogenesis of BWT is still unclear, but it is assumed to stem from the intralobarnephrogenic rests (ILNR) situated in the wall of the pelvicaliceal system [8]. According to the histologic findings, we hypothesis that tumor may extend into the ureter and bladder by mediating transitional cells of the urothelium (Figure 3A).

**Table 1:** Botryoid Wilms’ tumor (BWT) reported in the article

**Author(1st)/year**	**Region**	**Age**	**Gender**	**Side**	**Extension**	**Prognosis**
Reziciner/1970	European	6 years	M	R	Renal pelvis	Recurrence
Engel/1976	American	4 years	F	R	Renal pelvis	Unknown
Wicklund/1980	American	1 year	M	L	Renal pelvis	NED 2 years
Chiba/1980	Asian	1 year	F	R	Ureter	Unknown
Mahoney/1981	American	1 year	M	L	Renal pelvis	NED 2 years
Weinberg/1984	European	9 months	F	R	Renal pelvis	NED 4 years
Johnson/1987	American	8 years	M	R	Ureter	Unknown
Tunali/1987	Asian	4 years	M	L	Ureter	Unknown
Fu/1992	Asian	2 years	F	L	Renal pelvis	NED 2 years
Losty/1993	European	1 year	M	R	Bladder	NED
Niu/1993	Asian	4 months	M	L	Ureter	NED 2 years
Niu/1993	Asian	9 years	M	R	Renal pelvis	NED 10 months
Mitchell/1997	American	23 months	F	R	Bladder	NED 9 months
Honda/2000	Asian	1 year	M	R	Ureter	NED 5 years
Yanai/2005	Asian	3 years	M	R	Ureter	NED 4 years
Yanai/2005	Asian	2 years	M	R	Ureter	NED 9 months
Nagahara/2006	Asian	3 years	M	R	Bladder	NED 10 months
**Ceyla/2009**	Asian	4 years	F	L	Bladder	NED 6 months
Lamalmi/2010	European	14 months	M	R	duodenum	NED 2 years
Current case	Asian	4 years	M	R	Bladder/urethra	NED 2 years

F, Female; L, Left; M, Male; NED, No evidence of disease; R, Right.

Similar to our case, botryoid Wilms’ tumors with lesions were detected in only five cases [9-12]. This case has reported that parts of the necrotic tumor tissue then discharged from the urethra. The most common presenting symptom of BWT is gross hematuria, observed in approximately 25% of patients [9]. In this case, the patient had chief symptoms of gross hematuria and micturition pain. The exact pathogenesis of the micturition pain was not figure out. Parts of the necrotic tumor tissue or an intravesical tumor may obstruct the bladder neck and urethra, thus causing the symptom.

With difficulty in diagnosis of rare BWTs, it is important to consider differential diagnoses such as malignant rhabdoid tumor of kidney or xanthogranulomatous pyelonephritis. We applied CT to confirm the diagnosis of BWT in our patient. Imaging analysis demonstrated that tumors clearly extended from the renal pelvis into the ureter and bladder (Figure 2).

The treatment and prognosis of BWT should be no different from ordinary WT of similar stage and grade. Radical nephroureterectomy was the procedure of choice to avoid the risk of a recurrence in the ureteral stump. Preoperative adjuvant chemotherapy is also currently applied. After surgery, the patients should receive chemotherapy referring to the regimen of the National Wilms’ Tumor Study Group 5 (NWTS-5).

## Conclusions

In this paper, we presented our experience of diagnosis and treatment in Wilms’ tumor based on a special case in our hospital. In our opinion, the treatment and prognosis of botryoid Wilms’ tumor should be no different from ordinary Wilms’ tumor of similar stage and grade. The patients should receive chemotherapy referring to the regimen of the National Wilms’ Tumor Study Group 5 (NWTS-5). Whereafter, we summarized that gender, regional difference, and environmental factor may be important pathogenic elements of botryoid Wilms’ tumor by reviewing literatures.

## Consent

Written informed consent was obtained from the patient for publication of this case report and the accompanying images. Copies of the written consent are available for review upon request.

## Competing interests

The authors declare that there is no conflict of interest referring to this article.

## Authors’ contributions

GX and JH wrote the initial draft. YW and MX performed the surgery. YX performed the pathological examination. All authors read and approved the final manuscript.
